# Self-managed occupational therapy and physiotherapy for adults receiving inpatient rehabilitation (‘My Therapy’): protocol for a stepped-wedge cluster randomised trial

**DOI:** 10.1186/s12913-021-06462-9

**Published:** 2021-08-13

**Authors:** Natasha K. Brusco, Christina L. Ekegren, Nicholas F. Taylor, Keith D. Hill, Annemarie L. Lee, Lisa Somerville, Natasha A. Lannin, Derick Wade, Rania Abdelmotaleb, Libby Callaway, Sara L. Whittaker, Meg E. Morris

**Affiliations:** 1 Rehabilitation, Ageing and Independent Living (RAIL) Research Centre, School of Primary and Allied Health Care, Monash University, 47-49 Moorooduc Hwy, VIC 3199 Frankston, Australia; 2 La Trobe University Centre for Sport and Exercise Medicine Research, Plenty Road & Kingsbury Drive, 3086 Bundoora, Australia; 3 Alfred Health, 55 Commercial Rd, 3004 Melbourne, Australia; 4 Eastern Health, 5 Arnold St, 3128 Box Hill, Australia; 5 Cabrini Health, 154 Wattletree Rd, 3144 Malvern, Australia; 6 School of Physiotherapy, Monash University, 47-49 Moorooduc Hwy, VIC 3199 Frankston, Australia; 7 Department of Neuroscience, Monash University, Central Clinical School, 99 Commercial Rd, 3004 Melbourne, Australia; 8 Physiotherapy and Rehabilitation, Faculty of Health and Life Sciences, Oxford Brookes University, Headington Campus, OX3 0BP Oxford, United Kingdom; 9 School of Occupational Therapy, Monash University, 47-49 Moorooduc Hwy, VIC 3199 Frankston, Australia; 10 Healthscope ARCH, The Victorian Rehabilitation Centre, 499 Springvale Road, 3150 Glen Waverley, Australia

**Keywords:** Rehabilitation, Occupational Therapy, Physiotherapy, Physical Therapy, Intensity, Self-management, Implementation, Economic, Independence, Exercise

## Abstract

**Background:**

Ensuring patients receive an effective dose of therapeutic exercises and activities is a significant challenge for inpatient rehabilitation. My Therapy is a self-management program which encourages independent practice of occupational therapy and physiotherapy exercises and activities, outside of supervised therapy sessions.

**Methods:**

This implementation trial aims to determine both the clinical effectiveness of My Therapy on the outcomes of function and health-related quality of life, and cost-effectiveness per minimal clinically important difference (MCID) in functional independence achieved and per quality adjusted life year (QALY) gained, compared to usual care. Using a stepped-wedge cluster randomised design, My Therapy will be implemented across eight rehabilitation wards (inpatient and home-based) within two public and two private Australian health networks, over 54-weeks. We will include 2,160 patients aged 18 + years receiving rehabilitation for any diagnosis. Each ward will transition from the usual care condition (control group receiving usual care) to the experimental condition (intervention group receiving My Therapy in addition to usual care) sequentially at six-week intervals. The primary clinical outcome is achievement of a MCID in the Functional Independence Measure (FIM™) at discharge. Secondary outcomes include improvement in quality of life (EQ-5D-5L) at discharge, length of stay, 30-day re-admissions, discharge accommodation, follow-up rehabilitation services and adverse events (falls). The economic outcomes are the cost-effectiveness per MCID in functional independence (FIM™) achieved and per QALY gained, for My Therapy compared to usual care, from a health-care sector perspective. Cost of implementation will also be reported. Clinical outcomes will be analysed via mixed-effects linear or logistic regression models, and economic outcomes will be analysed via incremental cost-effectiveness ratios.

**Discussion:**

The My Therapy implementation trial will determine the effect of adding self-management within inpatient rehabilitation care. The results may influence health service models of rehabilitation including recommendations for systemic change to the inpatient rehabilitation model of care to include self-management. Findings have the potential to improve patient function and quality of life, and the ability to participate in self-management. Potential health service benefits include reduced hospital length of stay, improved access to rehabilitation and reduced health service costs.

**Trial registration:**

This study was prospectively registered with the Australian and New Zealand Clinical Trials Registry (ACTRN12621000313831; registered 22/03/2021, http://www.anzctr.org.au/Trial/Registration/TrialReview.aspx?id=380828&isReview=true).

## Background

Globally, inpatient rehabilitation costs are substantial. In the UK, there are 2.2 million NHS-funded inpatient rehabilitation admission across Complex Specialised, Specialist and Non-specialist Services annually, which cost the NHS £858 million (GBP 2018/19) [1, 2]. In the US, Medicare is the main insurer for inpatient rehabilitation within skilled nursing facilities [3] and intensive rehabilitation within hospital settings [3, 4]. There are 2.5 million funded skilled nursing facilities admissions [3] and 408,000 hospital inpatient rehabilitation admissions annually [4, 5] $28 billion (USD 2016) [4, 5] $8 billion (USD 2018) [$1.2 billion (AUD 2015/16) annually [6–8]. There is also evidence that the cost and demand for inpatient rehabili-tation is increasing [9]. This growth is thought to be driven by the ageing population, increasing survival fol-lowing acute illness and injury, greater comorbidity in patients, and higher expectations of recovery within the general population [9].

Rehabilitation is defined as “a process whereby a person who has continuing problems arising from an illness is helped both to reduce the extent of their difficulties and to use whatever personal strengths and skills they have, so that they can achieve goals of importance to them, both in the shorter term and, more importantly, in the longer term” [10]. Effective rehabilitation interventions include exercise, practice of tasks, education and self-management by the patient [11]. Clinical guidelines for general adult inpatient rehabilitation recommend three or more hours of therapy per weekday in addition to weekend therapy as tolerated [12]. Rehabilitation recommendations for specific cohorts, such as patients post-stroke, vary between one and a half and three hours per day for occupational therapy and physiotherapy participation [13, 14]. However, there is an evidence-practice gap and many services provide far less than the recommended amount [15–17]. Receiving less therapy than is recommended may be the reason patients may fail to achieve an optimal outcome in their recovery from injury, illness, or disease [18]. The low activity level in rehabilitation can also lead to patients feeling disempowered, bored and frustrated [19], where they spend most of the day sitting and lying down [20, 21].

While funding additional staff to increase the amount of supervised occupational therapy and physiotherapy could be an option [22], limited financial resources are often a barrier to implementation [23]. An alternative is to effectively increase the dose of inpatient rehabilitation by engaging patients in self-management through independent practice of occupational therapy and physiotherapy exercises and activities, outside of supervised therapy sessions.

My Therapy is a self-management program whereby the patient is educated and empowered to complete additional practice in-between therapist supervised sessions, during their episode of inpatient rehabilitation [24]. My Therapy involves goal-setting which is completed by both the patient and the therapist; patient education, motivation and empowerment; in addition to independent self-practice. While the My Therapy goals inform the individually tailored exercises and activities recommended by the therapist, it is the patient who decides which exercises and activities are practiced, and how much they are practiced each day.

While the definition of self-management in the literature is unclear and at times contentious [25], My Therapy defines self-management as a component of care which facilitates behaviour change and allows people to develop the skills to work with their problems or challenges, identify and contribute towards their own goals and ultimately become responsible for their own rehabilitation and health [26, 27]. Self-management includes education and advice about the condition, is built on the patient’s own goals and equips patients to make decision about their health [28]. Achieving self-practice within a self-management program is dependent on motivation, including intrinsic patient motivation and the therapists ability to motivate the patient, as well as the severity of the condition [29].

To differentiate My Therapy from general therapist advice and education regarding self-practice during inpatient rehabilitation and goal setting with the patient, My Therapy must also adhere to the following criteria (a) a written program, (b) therapist documentation of the program within the medical record, (c) include a feedback mechanism to the therapist (e.g., a paper based or smart device tick sheet), and (d) be actively monitored and progressed as clinically required. My Therapy is tailored to individual needs, recommended by the patient’s treating occupational therapist and physiotherapist after goal setting with the patient, and can be practiced within business hours, in the evenings or over the weekend.

A recent pilot study showed the implementation of My Therapy to be feasible in hospitalised older people with musculoskeletal conditions and frailty (n = 116) [24]. The pilot study led to around 100 min of extra weekly practice alongside usual care rehabilitation and the benefits occurred without additional staff or adverse events [24]. Compared to usual care, more than double the proportion of patients allocated to My Therapy achieved a minimal clinically important difference (MCID) in functional independence from admission to discharge [24], and it was also delivered safely in those with cognitive impairment.

The current protocol builds on this initial pilot study [24]. The project will scale-up implementation of My Therapy across inpatient and home-based rehabilitation wards in Australian public and private health networks, and evaluate the benefits and cost-effectiveness of My Therapy via a stepped-wedge cluster randomised trial design. This trial aims to determine the effectiveness of My Therapy on clinical outcomes of function and health-related quality of life, when implemented across four health networks. It will also determine the cost-effectiveness per MCID in functional independence achieved and per quality adjusted life year (QALY) gained compared to usual care; in addition to the cost of implementation. We hypothesise that My Therapy will result in improved functional independence and health-related quality of life during rehabilitation without adverse events (falls); and will be cost-effective to the health service due to a reduction in rehabilitation length of stay, compared to usual care alone.

## Methods/design

This protocol is reported in accordance with the Standard Protocol Items: Recommendations for Interventional Trials (SPIRIT) reporting guidelines [30] and the Consolidated Health Economic Evaluation Reporting Standards (CHEERS) [31]. A Project Steering Committee, comprising investigators, site clinicians and a consumer representative, will meet on a four-monthly basis to review trial progress. Any modifications to the trial protocol will be reported on the Australian and New Zealand Clinical Trials Registry (anzctr.org.au).

### Design

**Fig. 1:**
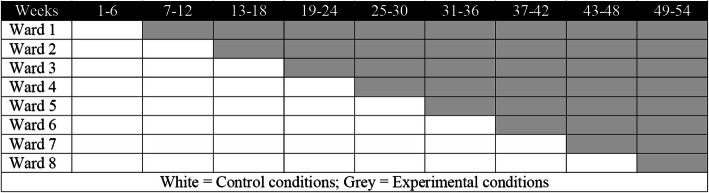
Timeline of program implementation

A stepped-wedge cluster randomised trial design will be conducted over 54 weeks, involving nine blocks of six-weeks duration (Fig. 1) [32]. Randomisation refers to the time when each ward crosses over from usual care to experimental conditions. Three months prior to trial commencement, a blinded researcher, not involved in the assessment or delivery of intervention, will randomise wards to the order of commencement of My Therapy using a computer-generated number sequence. Each ward will transition from the usual care condition (control group receiving usual care) to the experimental condition (intervention group receiving My Therapy in addition to usual care) sequentially at six-week intervals until all sites have crossed over to the experimental condition. Therapists and assessors will not be blind to group allocation and they will be informed of their upcoming cross over six weeks prior to cross over. This is due to the practical need to provide My Therapy education to the staff in the six weeks prior to commencing. In addition, patients will not be blind to group allocation due to the need to educate and engage patients in the My Therapy program.

### Setting and participants

The project will take place within eight rehabilitation wards (totalling over 200 rehabilitation beds) across four Victorian health networks (see anzctr.org.au for study sites). At one health service, the two wards will incorporate home-based bed-substitution; all others will be located within inpatient facilities. We will include all patients aged 18 years or older undergoing rehabilitation on the selected rehabilitation wards during the trial period, of any rehabilitation diagnosis. Patients who do not speak English and those with a cognitive impairment will not be excluded. We will exclude any patients without coverage under Australia’s universal health care program (Medicare).

### Participant recruitment

This trial will use an opt-out approach for consent [33]. Throughout the 54-week study period, all patients admitted to a participating ward will be provided with a participant information sheet on admission. The form will explain the research project, how participant information will be accessed and used and how to opt-out if patients do not wish for their information to be used in this project. An interpreter will be accessed for patients with inadequate English. Next of kin will be contacted for all patients with a documented cognitive impairment who are unable to understand project involvement; next of kin may choose to opt-out on behalf of the patient. The project data of any patients who choose to opt-out will not be collected by researchers.

### Usual care and intervention (Table 1)

**Table 1:** Description of experimental and control group conditions according to the template for intervention description and replication (TIDieR) [34]

	Intervention group under experimental conditions	Control group under usual care conditions
Brief name	My Therapy in addition to usual care rehabilitation	Usual care rehabilitation
Why	A higher dose of inpatient rehabilitation results in better patient and health service outcomes.	To provide a comparison with My Therapy.
What materials	In addition to usual care materials, patient information materials include a My Therapy explanation pack and an electronic or paper based My Therapy program. Additional materials include adjuncts to the recommended tasks and exercises such as written cognitive tasks, hand weights and resistance bands. Staff information materials include a My Therapy explanation pack and user guide.	Usual care materials may include exercise equipment, or equipment for practice of functional tasks, such as kitchen or bathroom facilities.
What procedures	On commencement of rehabilitation, the occupational therapist and physiotherapist will complete a full assessment of the patient and develop a rehabilitation plan that is guided by patient centred goals. Where it is deemed safe and appropriate, a sub-set of the supervised occupational therapy and physiotherapy exercises and activities will be provided to the patient to be practiced independently and outside of supervised sessions. To be classified as a self-management program, as is the case for My Therapy, it must include each of the following, (a) a written program, (b) therapist documentation of the program within the medical record, (c) a feedback mechanism to the therapist (e.g., a paper based or smart device tick sheet), and (d) will be actively monitored and progressed as clinically required. The exercises and activities will be delivered to patients via the online exercise prescription program, www.physiotherapyexercises.com (PTX). Following discussion and input from the patient, the treating clinicians will log into PTX, select the exercises and activities for each individual patient, and then send the My Therapy program to the patient’s own device (for example mobile phone, iPad, or laptop) via SMS or email, or the therapist will print out the My Therapy program as a booklet. While the My Therapy program will specify the number of repetitions and sets, the patient will decide what is done, how often and when. My Therapy can be updated as often as required. My Therapy will commence at the beginning of the rehabilitation admission. It will continue throughout the rehabilitation admission until the day of discharge, when appropriate and safe to do so.	On commencement of rehabilitation, the occupational therapist and physiotherapist will complete a full assessment of the patient and develop a rehabilitation plan that is guided by patient centred goals. The exercises and activities will be practiced with patients under therapist supervision. The program can be updated as required. It will commence at the beginning of the rehabilitation admission and continue throughout the rehabilitation admission until the day of discharge. Under usual care conditions, some therapists may provide self-management to some patients, representing heterogeneity of clinical practice. However, it is understood that prior to the My Therapy study at the participating sites, the provision of a self-management program (or components of a self-management program) was uncommon, clinician dependent and without systematic processes or monitoring. In this study, the provision of individual components of a self-management program is considered advice and education, not self-management. To be classified as a self-management program, as is the case for My Therapy, it must include each of the following, individual components, (a) a written program, (b) therapist documentation of the program within the medical record, (c) include a feedback mechanism to the therapist (e.g., a paper based or smart device tick sheet), and (d) be actively monitored and progressed and clinically required. The frequency of provision of self-management (as distinct from advice and education), under usual care conditions, will be assessed as a part of the Process Evaluation (Process Evaluation Protocol [36]).
Who provides	Registered occupational therapists and physiotherapists will complete the assessments, establish and progress the My Therapy program. They will be provided with study familiarisation, explanation and a user guide. All other members of the rehabilitation team, including nursing, medical and allied health assistants, will support the program through patient encouragement and conversation, however they will not progress exercises or supervise the practice.	Registered occupational therapists and physiotherapists will deliver usual care.
How provided	My Therapy is delivered to each patient by the occupational therapists and physiotherapists.	Usual care is individually tailored for each patient according to their rehabilitation goals and progressed throughout the rehabilitation admission.
Where (setting)	Independent practice of My Therapy can be completed in the patient’s room on the ward, the hallway, or other designated independent practice areas.	Most often completed in gyms or therapy rooms within rehabilitation hospitals.
When/how much (dose)	While a goal will be set for the amount of independent My Therapy practice, it is the patient who decides the number of sessions, the durations of the sessions and the number of repetitions.	Site-dependent, based on staff ratios and funding.
Tailoring	My Therapy is individually tailored for each patient according to their rehabilitation goals and progressed throughout the rehabilitation admission.	Usual care rehabilitation is individually tailored for each patient according to their rehabilitation goals and progressed throughout the rehabilitation admission.
Fidelity checking measures	Adherence and fidelity to the intervention will be assessed as a part of the Process Evaluation (Process Evaluation Protocol [36]).	Adherence and fidelity to usual care will be assessed as a part of the Process Evaluation (Process Evaluation Protocol [36]).

As part of usual care, a registered occupational therapist and physiotherapist employed by the health service will complete a full assessment of each patient, as per their routine procedures, and develop a rehabilitation plan that is guided by patient-centred goals. This is already routine practice at each of the participating sites. When wards transition to experimental conditions, all patients will continue with their usual rehabilitation plan; in addition, they will commence My Therapy. For My Therapy, the same occupational therapist and physiotherapist employed by the health service will provide patients with a sub-set of occupational therapy and physiotherapy exercises and activities to be practiced independently (where it is deemed safe and appropriate), outside of supervised sessions. These exercises and activities may be performed in the patients’ rooms on the ward or other designated independent practice areas. My Therapy will include (a) a written program, (b) therapist documentation of the program within the medical record, (c) include a feedback mechanism to the therapist (e.g., a paper based or smart device tick sheet), and (d) be actively monitored and progressed as clinically required.

The exercises and activities will be selected by the treating therapist from the available exercises within the online exercise prescription program, PTX found at www.physiotherapyexercises.com. Where a required exercise is not available, the therapist will upload additional customised exercises for use. Following discussion and input from the patient, the treating clinicians will log into PTX, select the exercises and activities for each individual patient, and then send the My Therapy program to the patient’s own device (for example mobile phone, iPad, or laptop) via SMS or email, or the therapist will print out the My Therapy program as a booklet.

The occupational therapist and the physiotherapist will collaborate to combine the discipline specific exercises and activities to ensure a co-ordinated approach. The recommended number of repetitions and sets of each exercise or task will be specified and can be updated by the occupational therapist and physiotherapist as often as required. While a goal will be set for the amount of independent practice, it is the patient who decides the actual number of sessions, the duration of the sessions and the number of repetitions that they complete, each day. This will be progressed as required throughout the rehabilitation admission.

Occupational therapists and physiotherapists will have regular daily contact with patients from Monday to Friday. Weekend occupational therapy and physiotherapy service provision varies from one health nextweek to the next and this ranges from no weekend service to a seven-day service. Additional materials will be provided free of charge to the patient. These include adjuncts to the recommended tasks and exercises, such as written cognitive tasks, adaptive equipment (e.g., long handled shoe horn; over-toilet frame), and therapy equipment (e.g., weights and resistance bands).

In the six weeks before crossover to the My Therapy intervention, occupational therapists and physiotherapists will be provided with a My Therapy explanation pack and a user guide, study familiarisation and education sessions, and interactive group discussions to co-design local implementation strategies. All other members of the rehabilitation team, including nursing, medical and allied health assistants, will attend the study familiarisation sessions and will be able to support the program through patient encouragement and conversation. However, they will not progress exercises or supervise the practice.

Where needed, there will be hospital/ward-based tailoring to adapt to the variety of settings, patient groups and therapy teams. For example, at the health service with home-based bed-substitution wards, the allied health assistant may deliver the paper-based My Therapy program to the patient and provide some education, when the occupational therapist or physiotherapist cannot do so in a timely manner. However, the allied health assistant will not supervise the My Therapy exercises, nor will they modify or progress the program. Adherence to the intervention will be assessed via the Process Evaluation Protocol [35].

**Fig. 2:**
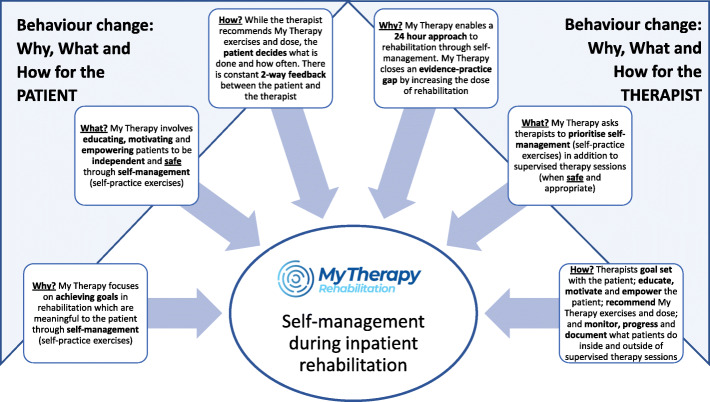
The Why, What and How of behaviour change for the patient and the therapist

Behaviour change by the patient and the therapist is key to successful implementation of My Therapy. Figure 2 describes the Why, What and How [36] of behaviour change for the patient and the therapist and these concepts will be core to the therapist education, prior to My Therapy implementation, as well as core to the My Therapy patient education provided by the therapists.

### Outcomes

#### Clinical evaluation

The primary clinical outcome is *functional independence*, which will be assessed using the Functional Independence Measure (FIM™) [37, 38]. The change in FIM™ from admission to discharge will be reported as the pro-portion of patients who achieve a MCID in FIM™ (22 points) [37]. The FIM™ is routinely recorded on admission and discharge from rehabilitation by accredited assessors, and will be used to report an overall score and sub-categories of motor and cognitive function.

Secondary outcomes include change in *health-related quality of life*, assessed using the EQ-5D-5L [39]; *rehabilitation length of stay*, reported as the number of overnight stays between the rehabilitation admission and discharge dates; *days at home up to 30 days after surgery (DAH30)* for any surgical patients [40, 41]; hospital re-admissions to the same health service one month (30 days) post-discharge (yes/no); discharge accommodation, categorised as home, transitional care, residential care, hospital, or death (no accommodation); need for post-discharge rehabilitation services (yes/no); and adverse events (falls), assessed as the number of falls and the number of serious falls during the rehabilitation admission, and these will be classified as occurring during or not during My Therapy participation.

#### Economic evaluation

For the economic evaluation, all cost data will be inflated to AUD$ 2021/22 (final year of data collection) using the consumer price index (CPI) (http://www.abs.gov.au/ausstats), and will take a health-care sector perspective, with a rehabilitation admission and 30-day re-admission time horizon. Discount rates will not be applied due to the limited time horizon. Six months after the end of the 2022 financial year (i.e., December 2022), all data will be available from the costing unit of each participating health service.

The primary economic outcome is the *incremental cost-effectiveness ratio (ICER)*, comparing the intervention and control groups using the health service cost, including 30-day re-admissions, as the numerator, and the utility data (quality adjusted life years (QALYs)) or effectiveness data (FIM™) as the denominator. A secondary economic outcome is the *cost of implementation* which will be recorded to inform future adoption and scaling of My Therapy and to understand the cost of the My Therapy intervention, independent of the health service costs for each rehabilitation patient (details in data analysis section).

### Data collection and management

All data, including the primary outcome, will be obtained from medical records, hospital incident reporting systems, or via hospital costing units. Study data will be collected and managed using REDCap (Research Electronic Data Capture) electronic data capture tools hosted at Monash University and managed by Helix [42, 43]. EDCap is a secure, web-based application designed to support data capture for research studies, providing (1) an intuitive interface for validated data entry; (2) audit trails for tracking data manipulation and export procedures; (3) automated export procedures for seam- less data downloads to common statistical packages; and (4) procedures for importing data from external sources. On each ward, a designated allied health assistant, employed by the health service will enter data from participants’ medical record into a custom-built REDCap database. The site coordinator will review the accuracy of the study data from a random selection of 20% of participants each week. The site coordinator will randomly sample based on the availability of the medical records at the time of data review.

Every six weeks, each site’s coordinator will extract data recorded about falls from the hospital reporting system. Falls data will be reviewed by the Independent Data Safety and Monitoring Committee (DSMC) [44]. The DSMC will comprise of two experienced independent researchers who have no vested interest in the outcome of the trial and they will remain blind to ward allocation. We have prespecified a falls event rate *>* 10% over the normal upper limit, based on 12 months of historical falls data from the participating wards, as requiring intervention by the trial steering committee. Any harm suffered from participation in the trial will be managed by the health service who provided rehabilitation care.

### Data Analysis

#### Sample size calculation

The sample size calculation is based on the proportional change in primary outcome measure (the FIM™) in the published My Therapy pilot study, which found that double the proportion of patients in the My Therapy group (22%) achieved a MCID in function (FIM) from admission to discharge, compared to usual care (10%) [24]. The sample size is based on a two-tailed alpha of 0.05, power of 0.99, as well as an intraclass correlation coefficient (ICC) of 0.005 (established from a previous rehabilitation study [20]). Using these parameters, the total sample size required is 2,160 patients. With an average of 25 beds per ward, length of stay of 18.5 days [45] and a data collection period of 54 weeks, each rehabilitation ward will contribute around 485 patients. Allowance has been made for this to be reduced to 340 when an opt-out rate of 30% is assumed. If each of the eight rehabilitation wards contributes 340 patients across the 54 weeks of data collection, the total of 2,716 will exceed the sample size requirement for the primary outcome measure of the proportion of people who achieve a MCID in FIM™. The sample size calculation allows for additional planned bed closures over the Christmas period and censoring of patients already admitted to the ward at the point of transition from the control to the experimental period, as these patients will be exposed to both conditions and will be censored to avoid data contamination.

#### Statistical analyses

Analyses will only include data from patients who were admitted and discharged during the ward’s control condition period or experimental condition period. Patients still admitted to the rehabilitation wards at the end point of the study will not be included. It is expected that due to the step-wedged design, this will equally exclude long-stayers in the control and experimental condition periods. Each analysis will use individual patient-level data, clustered within the ward, and will use the length of time (in weeks) of experimental exposure as an effect modifier [32]. Length of time of exposure to the experimental condition is an important consideration when an intervention may take time to settle into usual practice and where there may be a cumulative effect [32]. Analyses will be conducted on an intention-to-treat basis.

The analyses will compare the FIM™ and the health-related quality of life utility index score between the control and the intervention groups. Health-related quality of life scores from the EQ-5D-5L will be converted from raw scores to a utility index based on an Australian population [46, 47]. The proportion of people who achieve a MCID of 22 points in FIM™ [35, 37] will be analysed using mixed effects logistic regression, and the change in FIM™ score and utility index will be ana-lysed using mixed effects linear regression. Length of stay will also be analysed via a linear regression model.

While not powered for a secondary analysis, there will be an exploratory secondary analysis of the same outcome measures within each of the clusters and for the two home-based bed-substitution wards. Other secondary outcomes will be compared between groups using univariable analyses. All outcomes will be reported with 95% confidence intervals and analyses will assume a significance of *p* < 0.05.

For the economic evaluation, the primary outcome will be presented as cost-effectiveness and cost-utility analyses, reporting (i) the cost per MCID in FIM™ achieved; and (ii) the cost per QALY gained. Costs will include the rehabilitation length of stay, acute transfers within the rehabilitation stay and unplanned re-admissions within the 30 days post discharge. The QALYs will be calculated using the utility index scores.

The mean cost difference will be determined between the two groups using a linear regression model [48]. Incremental cost-effectiveness ratios will be determined for both the FIM™ and utility index using the cost as the numerator, and the utility or effectiveness data as the denominator. All analyses will use individual patient-level data, will be clustered within the ward, and will use the length of time (in weeks) of experimental exposure as an effect modifier [32]. The ICERs for the cost per MCID achieved will be analysed using mixed effects logistic regression, and the ICERs for the cost per QALY gained will be analysed using mixed effects linear regression. Confidence intervals around the individual ICERs for utility and effectiveness will be calculated using the bootstrap method with 5,000 repetitions [49]. Individual ICERs will use the central limit theorem to generate the confidence ellipses, and the cost effectiveness acceptability curves (CEACs) [49] to inform the probability that My Therapy is less costly and more beneficial compared with usual care alone, with a $50,000 per QALY gained [50] threshold to determine cost-effectiveness.

A cost-analysis will be utilised to determine the cost of implementation. Data will be presented within two time periods; (i) the preparation period while under usual care conditions as well as the three months prior to commencing My Therapy (e.g., materials purchased and staff education sessions), and (ii) the implementation period while under experimental conditions (from commencement of My Therapy through to the end of the inpatient data collection indicating business as usual). Periods (i) and (ii) together will represent the full cost of My Therapy implementation (i.e., preparation, implementation and transition to business-as-usual conditions), whereas just period (ii) will represent the costs under business-as-usual conditions (i.e., just implementation and transition to business-as-usual conditions).

As each ward will have a different time length for the preparation period and the implementation period, depending on their point of randomisation, costs will be presented per patient (total costs divided by the total number of patients who participate in My Therapy), and per ward (total costs divided by the total number of wards; n = 8) using independent *t*-tests (mean, standard deviation). Resource utilisation will be reported as real and in-kinds costs and these will include exercise materials purchased specifically for My Therapy (reported as market rate at the time of purchase); education for staff and patients, site coordinator time, marketing and communication, as well as therapist time to prescribe and progress My Therapy (staff costs based on EBA rates for Health Professionals) [51]. Costs will not include incidental nursing, medical or other staff time spent encouraging patient participation in My Therapy.

### Dissemination

Each participating health service will be provided a summary of overall findings at the conclusion of all data collection. Scientific papers will be written to address the research aims and these will be submitted to high-impact peer-reviewed journals for publication. Results will also be disseminated via a dedicated website (mytherapyrehab.com.au). A community of practice will be developed and dedicated to supporting patients, clinicians, researchers, and health networks to encourage patient self-management as part of rehabilitation. While currently in development, it is proposed that the community of practice will be centred around three key attributes: the community (patients, clinicians, researchers and health networks), the practice (that is, mytherapyrehab.com.au as the environment) and the domains (the goal, identity and purpose of the community of practice) [52, 53]. Members of the community of practice will be mentored by members of established communities of practice, including that of the Australian Rehabilitation and Assistive Technology Association (see www.arata.org.au).

## Discussion

This multi-site stepped-wedge cluster randomised trial will scale-up the implementation of the previously-tested My Therapy program across rehabilitation wards in public and private hospitals in Australia. Both clinical effectiveness and program cost effectiveness will be evaluated. A feature of My Therapy is its promotion of patient self-management, which in turn allows people to develop the skills to work with their problems or challenges, identify and contribute towards their own goals and ultimately be responsible for their own rehabilitation and health [26, 27]. My Therapy may also provide unintended benefits such as reducing patient disempowerment, boredom and frustration during rehabilitation [19].

## Limitations

While My Therapy aims to improve patient outcomes by increasing the therapy delivered, it does not attempt to differentiate the relative contribution of increased time dedicated to independent practice and the time spent with the therapist. In addition, My Therapy does not evaluate whether skilled therapists can teach a patient to practice safely, independently, and often. Future trials will need to ascertain how practice and therapy independently contribute to patient outcomes; where practice is the amount of time performing an exercise, task or activity [54], and therapy is the time spent with a professionally qualified therapist who educates patients, co-designs their goal-directed program, teaches them how to perform an activity, gives feedback, helps patients to practice and structures a learning environment [54], My therapy also includes teaching patients how to undertake an activity independently and enabling them to implement what they have learned in daily life [54].

There are also practical and operational issues which may potentially impact study procedures and outcomes. Each of these has been carefully considered, with *a priori* mitigation strategies put in place. Within trial sites, independent therapy prescription may already be practiced, to some extent, as part of usual care conditions. As such, there may be variability in clinicians’ receptiveness to implementing My Therapy and potential differences in effect sizes across trial sites. While My Therapy is still expected to differ substantially to usual practice, a full process evaluation (described in the Process Evaluation Protocol [36]). will be conducted to better understand these contextual factors. Another consideration relates to the inclusion of two ‘virtual’ home-based bed-substitution rehabilitation wards. While not initially planned, this change in practice at one of the included health networks presents a good opportunity for sub-group analyses, comparing the impact of My Therapy in traditional inpatient wards to home-based wards, which are becoming increasingly common in Australian hospitals.

## Conclusions

The My Therapy implementation trial will determine the effect of adding self-management within inpatient rehabilitation care. The results may influence health service models of rehabilitation including recommendations for systemic change to the inpatient rehabilitation model of care to include self-management. Findings have the potential to improve patient function and quality of life, and the ability to participate in self-management. Potential health service benefits include reduced hospital length of stay, improved access to rehabilitation and reduced health service costs.

## Data Availability

The datasets generated and/or analysed during the current study will not be publicly available as the Ethical Review Board approval was obtained for public sharing and presentation of data on a group-level only. However, individual-level data may be available from the corresponding author on reasonable request noting that this will require separate ethics approval for the dissemination and use of the data.

## Abbreviations

EQ-5D-5L: EuroQOL 5 Dimensions, 5 Level
FIM: Functional Independence Measure
ICC: Intraclass correlation coefficient
ICER: Incremental cost effectiveness ratio
MCID: Minimally clinically important difference
NHMRC: National Health and Medical Research Council
QALYs: Quality Adjusted Life Years
