# Correction to: Self-managed occupational therapy and physiotherapy for adults receiving inpatient rehabilitation (‘My Therapy’): protocol for a stepped-wedge cluster randomised trial

**DOI:** 10.1186/s12913-021-07002-1

**Published:** 2021-09-17

**Authors:** Natasha K. Brusco, Christina L. Ekegren, Nicholas F. Taylor, Keith D. Hill, Annemarie L. Lee, Lisa Somerville, Natasha A. Lannin, Derick Wade, Rania Abdelmotaleb, Libby Callaway, Sara L. Whittaker, Meg E. Morris

**Affiliations:** 1 Rehabilitation, Ageing and Independent Living (RAIL) Research Centre, School of Primary and Allied Health Care, Monash University, 47-49 Moorooduc Hwy, Frankston, VIC 3199 Australia; 2 La Trobe University Centre for Sport and Exercise Medicine Research, Plenty Road & Kingsbury Drive, Bundoora, 3086 Australia; 3 Alfred Health, 55 Commercial Rd, Melbourne, 3004 Australia; 4 Eastern Health, 5 Arnold St, Box Hill, 3128 Australia; 5 Cabrini Health, 154 Wattletree Rd, Malvern, 3144 Australia; 6 School of Physiotherapy, Monash University, 47-49 Moorooduc Hwy, Frankston, VIC 3199 Australia; 7 Department of Neuroscience, Monash University, Central Clinical School, 99 Commercial Rd, Melbourne, 3004 Australia; 8 Physiotherapy and Rehabilitation, Faculty of Health and Life Sciences, Oxford Brookes University, Headington Campus, Oxford, OX3 0BP UK; 9 School of Occupational Therapy, Monash University, 47-49 Moorooduc Hwy, Frankston, VIC 3199 Australia; 10 Healthscope ARCH, The Victorian Rehabilitation Centre, 499 Springvale Road, Glen Waverley, 3150 Australia

**Correction to: BMC Health Serv Res 21, 811 (2021)**

**https://doi.org/10.1186/s12913-021-06462-9**

Following publication of the original article [1], the author noted a small number of corrections:

1. In the first paragraph of the Background section, some content is missing due to a typesetting error. The updated first paragraph is given below and the missing part has been highlighted in **bold typeface**.

Globally, inpatient rehabilitation costs are substantial. In the UK, there are 2.2 million NHS-funded inpatient rehabilitation admission across Complex Specialised, Specialist and Non-specialist Services annually, which cost the NHS £858 million (GBP 2018/19) [1, 2]. In the US, Medicare is the main insurer for inpatient rehabilitation within skilled nursing facilities [3] and intensive rehabilitation within hospital settings [3, 4]. There are 2.5 million funded skilled nursing facilities admissions [3] and 408,000 hospital inpatient rehabilitation admissions annually [4, 5] **which respectively cost Medicare** $28 billion (USD 2016) [3] **and** $8 billion (USD 2018) [**4,5]. In Australia, there are half a million public and private rehabilitation hospital admissions per year [6–8], with the 91,000 public admissions costing the public health care system** $1.2 billion (AUD 2015/16) annually [6,7,8]. There is also evidence that the cost and demand for inpatient rehabilitation is increasing [9]. This growth is thought to be driven by the ageing population, increasing survival following acute illness and injury, greater comorbidity in patients, and higher expectations of recovery within the general population [9].

2. In Table 1 – The Process Evaluation Protocol should be referred to as Reference 35, instead of Reference 36.

3. In the Data collection and management section, the third sentence should start with “REDCap” instead of “EDCap”.

4. In the second paragraph of the Statistical analyses part in the Data Analysis section, the reference in one sentence needs to be corrected from [35,37] to [37,38]. The updated sentence should be:

The proportion of people who achieve a MCID of 22 points in FIM™ **[37,38]** will be analysed using mixed effects logistic regression, and the change in FIM™ score and utility index will be analysed using mixed effects linear regression.

The original article [1] has been corrected.
